# Medical Department of the University

**Published:** 1893-03

**Authors:** 


### MEDICAL DEPARTMENT OF THE UNIVERSITY.

---

THE SIXTY-FIRST COMMENCEMENT OF THAT INSTITUTION WAS HELD AT AUGUSTA, GA.

---

The sixty-first annual commencement of the Medical Department of the University of Georgia was held in Masonic hall. Chancellor Boggs, of the University, presided and presented the diplomas to a graduating class of thirty-two young doctors. The annual address was delivered by Rev. J. T. Plunket, of Augusta. The salutatory address was delivered by Dr. W. C. Lyle, of Carrollton, Ga., and the valedictory by Dr. W. W. Pilcher, of Norwood. The graduating class is composed of the following gentlemen:

George R. Maner, N. J. Coker, W. C. Leary, W. Wyan Pilcher, William C. Lyle, J. E. Norton, T. W. Ellis, J. H. Greene, J. C. C. LeHardy, H. L. Martin, Joel W. Waym, J. H. Self, H. H. Townes, I. J. Burch, J. L. Kennedy, Charles Meldrim, George W. Mountain, H. Smith, M. M.

Conners, A. L. Ray, A. J. Deas, James Mayhough, J. R. Autman, J. E. Moon, J. H. Williams, Wyatt C. Hatcher T. A. Powell, John C. Childs, H. C. Doughty, F. E. Lothridge, R. J. Videtto, C. N. Nix. The honors of the class were awarded as follows:

First honor, Dr. W. C. Lyle, of Carrollton, Ga.; second honor, Dr. Henry Campbell Doughty, Augusta, Ga.

